# Longitudinal relationships between Korean medical students’ academic performance in medical knowledge and clinical performance examinations: a retrospective longitudinal study

**DOI:** 10.3352/jeehp.2025.22.18

**Published:** 2025-06-10

**Authors:** Yulim Kang, Hae Won Kim

**Affiliations:** 1 Center for Educational Evaluation, Yonsei Donggok Medical Education Institute, Yonsei University College of Medicine, Seoul, Korea; 2 Department of Medical Education, Yonsei University College of Medicine, Seoul, Korea; The Catholic University of Korea, Korea

**Keywords:** Academic performance, Educational measurement, Longitudinal study, Medical student, Republic of Korea

## Abstract

**Purpose:**

This study investigated the longitudinal relationships between performance on 3 examinations assessing medical knowledge and clinical skills among Korean medical students in the clinical phase. This study addressed the stability of each examination score and the interrelationships among examinations over time.

**Methods:**

A retrospective longitudinal study was conducted at Yonsei University College of Medicine in Korea with a cohort of 112 medical students over 2 years. The students were in their third year in 2022 and progressed to the fourth year in 2023. We obtained comprehensive clinical science examination (CCSE) and progress test (PT) scores 3 times (T1–T3), and clinical performance examination (CPX) scores twice (T1 and T2). Autoregressive cross-lagged models were fitted to analyze their relationships.

**Results:**

For each of the 3 examinations, the score at 1 time point predicted the subsequent score. Regarding cross-lagged effects, the CCSE at T1 predicted PT at T2 (β=0.472, P<0.001) and CCSE at T2 predicted PT at T3 (β=0.527, P<0.001). The CPX at T1 predicted the CCSE at T2 (β=0.163, P=0.006), and the CPX at T2 predicted the CCSE at T3 (β=0.154, P=0.006). The PT at T1 predicted the CPX at T2 (β=0.273, P=0.006).

**Conclusion:**

The study identified each examination’s stability and the complexity of the longitudinal relationships between them. These findings may help predict medical students’ performance on subsequent examinations, potentially informing the provision of necessary student support.

## Graphical abstract

[Fig f2-jeehp-22-18]

## Introduction

### Background/rationale

A rigorous, multifaceted assessment of medical students’ knowledge and skills is critical for implementing competency-based medical education, which necessitates regular and comprehensive assessments [1]. Different examinations are used during medical students’ clinical years to assess their academic achievement in relevant domains, such as comprehensive subject examinations (e.g., the National Board of Medical Examiners’ subject exams) and clinical performance examinations (CPXs). Another assessment tool frequently used in undergraduate medical education is the progress test (PT), a curriculum-independent test of clinical knowledge conducted in a repetitive, longitudinal manner [2]. The former two assessments are frequently used for summative purposes, while the latter is used for both formative and summative purposes depending on the institutional context. In the assessment domain, subject examinations and PTs focus on medical knowledge, while CPXs address clinical skills. Several studies have investigated student achievement in these examinations and its relationship with clerkship characteristics [3-5]. However, limited research has addressed the relationships between examinations, particularly using a longitudinal design. A single-timepoint analysis of individual examination scores cannot demonstrate the complex interplay of different examinations in measuring students’ competence. Thus, it is not possible to determine whether each examination captures student learning growth over time, or whether one examination predicts future outcomes in other examinations. As it is important to identify whether medical students’ knowledge and skills evolve over time, it is necessary to examine how different examinations individually and interactively contribute to their measurement.

### Objectives

This study examined the longitudinal relationships among medical students’ academic performance in 3 major examinations, each of which was conducted multiple times: comprehensive clinical science examinations (CCSEs), CPXs, and PTs. In doing so, it identified changes in students’ performance and the interconnections among these assessment tools. The specific research questions related to (1) each examination’s stability over time, and (2) the relationships among the examinations over time.

## Methods

### Ethics statement

The Institutional Review Board (IRB) of Severance Hospital, Yonsei University Health System, approved this study as an IRB-exempt study, considering that it did not include personally identifiable information and utilized educational data obtained from standard educational settings (IRB no., 4-2025-0058). Therefore, the requirement for informed consent was waived.

### Study design

This study employed a retrospective, longitudinal design using data collected at 3 time points over 2 years to examine the relationships between medical students’ performance on examinations assessing medical knowledge and their clinical performance.

### Setting

This study was conducted at Yonsei University College of Medicine (YUCM) in Korea. At YUCM, the undergraduate medical education curriculum consists of 2 years of pre-medical studies and 4 years of the medical curriculum; the latter consists of preclinical and clinical phases (2 years each). During the clinical phase, students’ medical knowledge and skills are assessed multiple times through 3 comprehensive examinations, which were the focus of the study. In this study, we utilized the assessment data of a student cohort in their clinical years, collected 3 times between 2022 and 2023: Time 1 in November 2022 (third year), Time 2 in July to August 2023 (fourth year), and Time 3 in November 2023 (fourth year). No specific educational interventions were used in this study.

### Participants

This study targeted medical students in their third year in 2022 who progressed to their fourth year in 2023. We included those who had participated in 3 types of examinations, each conducted 2 to 3 times. To ensure data continuity, students with missing records for any of the 3 examinations were excluded, and the analysis used only complete datasets. This exclusion criterion was applied to minimize any potential bias from the inappropriate imputation of missing values, and to enhance the findings’ validity and reliability.

### Variables

This study analyzed 3 variables reflecting medical students’ academic performance: scores on 2 medical knowledge examinations and 1 CPX.

### Data sources/measurement

#### Comprehensive clinical science examination

CCSE scores administered by the Korea Association of Medical Colleges were used to measure medical students’ achievement in the medical knowledge domain. The CCSE’s results are used for summative purposes at YUCM, which are reflected in deciding whether students pass or fail specific courses. The CCSE consists of 320 multiple-choice questions on medical knowledge in various disciplines, including internal medicine, surgery, pediatrics, obstetrics and gynecology, and psychiatry. The current analysis used the percentage of correct scores, with CCSE data collected from T1 to T3.

#### Clinical performance examination

CPX scores were used to comprehensively measure clinical skill performance. The CPX consists of 9 stations with standardized patients, where students record patient histories, perform physical examinations, and provide patient education, as well as 1 station for examination of clinical skills in a structured format. Trained standardized patients were provided by the CPX Consortium of Medical Schools in Seoul and Gyeonggi-do, Korea. At YUCM, the CPX is used for summative purposes, and students must pass this examination based on certain criteria. This study’s analysis includes a total score on a 100-point scale, with CPX data collected at T1 and T2.

#### Progress test

PT scores were used as another measure of comprehensive medical knowledge. Progress testing in medical education is a repetitive, longitudinal approach to assess medical students’ knowledge acquisition and retention. The YUCM has introduced progress testing since 2014, and faculty members of the institution have been developing test items. The PT consisted of 150 multiple-choice questions related to clinical phenotypes that are essential for medical graduates. The test blueprint, consisting of these essential clinical phenotypes, serves as a basis for developing and selecting test items, ensuring that items have similar characteristics across tests. At YUCM, PTs are conducted 3 times during the academic year—in March, July, and November—resulting in at least 12 tests for each student during the curriculum’s medical phase. The PT results are used for formative purposes to provide feedback on students’ progress in accumulating and applying clinical knowledge over time. PT data were collected at T1 through T3, and the percentage of correct answers was analyzed.

All data are presented in Dataset 1.

### Bias

Selection bias is less likely in this study since we targeted the entire class, which was in its third year in 2022 and progressed to its fourth year in 2023.

### Study size

This study retrospectively analyzed data collected from normal educational practices; thus, the sample size was not calculated a priori.

### Statistical methods

All statistical procedures were performed using R software ver. 4.4.3 (https://www.r-project.org/). Specifically, the descriptive statistics for the examination scores at each time point were analyzed to explore the data’s overall distribution and trends. Second, Pearson correlation analysis was conducted to examine the relationships among the CCSE, CPX, and PT scores. Third, an autoregressive cross-lagged model (ARCLM) was applied to investigate a variable’s stability over time, and the directional relationships between variables at 2 to 3 time points. The ARCLM accounts for one variable’s influence on its future state (autoregressive effects), and one variable’s influence on another variable’s future state (cross-lagged effects) [6].

Eight competing models were developed (Table 1) and goodness-of-fit indices were compared to select the best model. The comparative fit index (CFI), Tucker-Lewis index (TLI), and standardized root-mean square residual (SRMR) were used. Any CFI and TLI values of 0.9 and above and an SRMR value of 0.08 and below were considered acceptable. Comparisons between models were performed using the *χ*^2^ goodness-of-fit statistic and the ∆CFI value, which is not sensitive to the sample size. ∆CFI values greater than 0.01 were deemed statistically significant. All ARCLM analyses were conducted with the R package ‘*lavaan*’ [7], and Supplement 1 presents the R code for the final model analysis.

## Results

### Participants

Of the 120 students, 112 were included in the final analysis. Participants’ demographic information, such as age and sex, was not included because this study obtained ethical approval to use pre-collected data without personal information.

### Main results

Table 2 presents the study variables’ descriptive statistics. Overall, scores for each of the 3 examinations increased over time.

Table 3 illustrates the correlations among the study variables at the 3 time points, all of which were statistically significant.

Table 4 presents the sequential validation results for the ARCL models. As the ∆CFI values did not exceed 0.01 despite significant differences in the *χ*^2^ statistic in some comparisons, the path and error covariance invariances over time were considered to have been identified. Therefore, model 8 was selected as the final model.

**Fig. 1.**
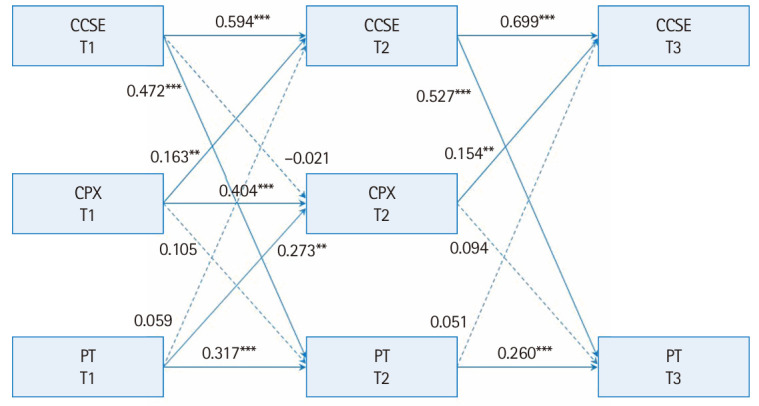
Results of the autoregressive cross-lagged model among the comprehensive clinical science examination (CCSE), clinical performance examination (CPX), and progress test (PT). All coefficients presented are standardized regression coefficients. Dashed lines indicate non-significant estimates. ^**^P<0.01. ^***^P<0.001.

Fig. 1 presents the autoregressive cross-lagged model results for the CCSE, CPX, and PT scores. Solid lines indicate significant paths and dashed lines indicate non-significant paths. The autoregressive analysis revealed stability among the 3 variables over time: CCSE, β=0.594–0.699, P<0.001; CPX, β=0.404, P<0.001; and PT, β=0.260–0.317, P<0.001. The cross-lagged analysis revealed that the CCSE at T1 significantly predicted PT at T2 (β=0.472, P<0.001), while its prediction of CPX at T2 was not significant. The CCSE at T2 significantly predicted PT at T3 (β=0.527, P<0.001). The CPX at T1 significantly predicted the CCSE at T2 (β=0.163, P=0.006); however, the prediction of PT at T2 was not significant. The CPX at T2 also predicted the CCSE at T3 (β=0.154, P=0.006), but not the PT at T3. Further, the PT at T1 significantly predicted the CPX (β=0.273, P=0.006), but not the CCSE, at T2; the PT at T2 did not significantly predict the CCSE at T3.

## Discussion

### Key results

This study examined the longitudinal stability and relationships among medical students’ academic performance on the CCSE, CPX, and PT. All 3 examination scores demonstrated significant stability over time, with cross-lagged effects indicating relationships at certain time points. The CCSE consistently revealed a significant predictive relationship with the PT, while the CPX significantly predicted the CCSE. In contrast, the PT only exhibited a significant predictive relationship with the CPX, while no significant predictive relationship with the CCSE was observed.

### Interpretation

This study’s findings demonstrate the longitudinal stability and interrelationships among clinical phase medical students’ key examination scores. Significant autoregressive effects were observed across all 3 examinations, indicating that students’ performance on each examination remained consistently stable over time.

The cross-lagged effect analysis demonstrated that the CCSE significantly predicted PT performance, suggesting that students who performed well on the CCSE were likely to maintain high performance on subsequent PTs. This finding suggests that comprehensive medical knowledge as assessed by the CCSE may contribute to promoting student knowledge’s long-term growth, which is the intended purpose of PTs. Furthermore, the CPX was found to predict CCSE performance, indicating that clinical performance capabilities may enhance outcomes in knowledge-based assessments, such as CCSE. This finding may be understood from the experiential learning theory perspective [8], as concrete experiences of engaging in clinical scenarios and problem-solving processes may promote the conceptualization and acquisition of theoretical knowledge. In contrast, the PT only significantly predicted the CPX, while its predictive effect on the CCSE was not statistically significant. Given the PT’s predominantly formative characteristics at the institution, aimed at monitoring longitudinal knowledge development and providing timely feedback [9], the PT may primarily support learning reinforcement and retention, partially contributing to improving clinical performance abilities. However, its impact on such high-stakes knowledge assessments as the CCSE may be less significant, as preparation strategies for the test may influence them.

### Comparison with previous studies

This study’s results are partially consistent with those of previous studies, offering important insights into the direction and role of interactions among examinations. The observed autoregressive stability over time in key examination scores during the clinical phase aligns with earlier studies that reported the longitudinal stability of academic achievement [10,11]. Specifically, the finding that CCSE predicts PT supports prior research suggesting the formative role of PTs in the long-term reinforcement of learners’ existing knowledge bases and the promotion of learning through feedback [12]. Similarly, the predictive relationship between the CPX and CCSE aligns with the educational value of experiential and clinical learning, which fosters the internalization of theoretical knowledge and enhances learning motivation [13]. Furthermore, the non-significant relationship between PT and CCSE is consistent with previous findings suggesting that as a low-stakes assessment, the PT is better suited to support learning and promote self-directed learning rather than predicting outcomes on high-stakes examinations; this reflects differences in the assessments’ purpose and utilization [14,15].

### Limitations

First, the sample size was relatively small, which could have limited the statistical power for identifying significant relationships among the study variables. Second, the 3 examinations used in this study were not standardized. Therefore, the difficulty of the examination may not have been identical across time points, which may have affected their achievement levels. Third, this analysis allowed unequal intervals between measurement points, reflecting the academic timeline. Although the intervals were not identical, each time point was set according to the progression of the curriculum, maintaining temporal ordering, and the model demonstrated an acceptable level of fit. Nonetheless, potential effects of the interval differences should be considered when interpreting the estimates, and future research may explore time-weighted modeling to achieve more refined results. Fourth, demographic information of the participants was not included in the analysis. Adjusting for the influence of demographic factors, such as age and sex, may allow a more accurate interpretation of the interrelationships among examination performance. Fifth, it was not possible for us to exclude the influence of systemic factors that could have affected the academic performance of this student cohort, such as the COVID-19 (coronavirus disease 2019) pandemic.

### Generalizability

The study’s generalizability may be limited by its analysis of a single cohort at one medical school, which was inevitable in part because of the study design, which required the sample to undergo the same examinations. Nonetheless, the present findings may be generalizable to other medical schools in Korea and globally, since the examinations used in this study are common assessment tools in undergraduate medical education. Given that the CCSE is a nationwide examination held by the Korea Association of Medical Colleges and that a regional consortium develops clinical scenarios for the CPX, these findings may have similar implications in other institutions that utilize these tools in Korea. Moreover, comprehensive examinations addressing subject knowledge and clinical competence are widely used worldwide, which may contribute to the study findings’ generalizability.

### Suggestions

Further studies with larger sample sizes, including participants from different institutional contexts, may enhance the results’ dependability and generalizability. The assessment tools shared across institutions may enable further research. Additionally, investigating the longitudinal stability of scores in each clinical discipline and the interrelationships among them can be advantageous in understanding whether students’ learning in different subject domains progresses similarly. Furthermore, extending the time points to include the Korean Medical Licensing Examination results would be beneficial for determining whether examinations during medical school predict critical outcomes of medical education.

Considering the complex interrelationships between examinations over time, educators may use examination scores at a specific time point as one piece of information to identify students who may benefit from academic support until the subsequent examination.

### Conclusion

The findings suggest that the results of 3 examinations frequently used to assess clinical-phase medical students exhibit both longitudinal stability and complex relationships. Therefore, medical educators may benefit from using examinations for different purposes and assessment domains to better understand clinical students’ academic developmental trajectories and support student success, as necessary.

## Figures and Tables

**Figure f2-jeehp-22-18:**
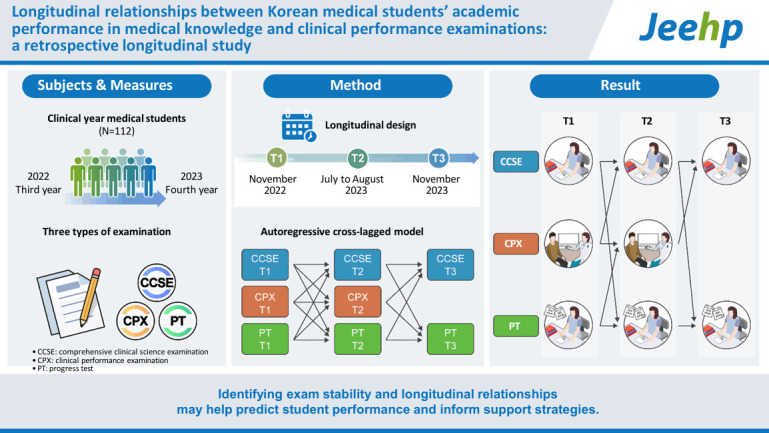


**Table 1.** Development of 8 competing research models for sequential validation

Variable	Model	Description
Unconstrained	Model 1	An unconstrained baseline model
Autoregressive path invariance	Model 2	A model in which the autoregressive coefficients for CCSE are equally constrained in model 1
	Model 3	A model in which the autoregressive coefficients for PT are equally constrained in model 2
Cross-lagged path invariance	Model 4	A model in which the cross-lagged coefficients from CPX to PT are equally constrained in model 3
	Model 5	A model in which the cross-lagged coefficients from PT to CCSE are equally constrained in model 4
	Model 6	A model in which the cross-lagged coefficients from CCSE to PT are equally constrained in model 5
	Model 7	A model in which the cross-lagged coefficients from CPX to CCSE are equally constrained in model 6
Error covariance invariance	Model 8	A model in which the error covariances between CCSE and PT are equally constrained in model 7

CCSE, comprehensive clinical science examination; PT, progress test; CPX, clinical performance examination.

**Table 2.** Assessment scores’ descriptive statistics over time

Variable	Mean±SD	Median	Min	Max	Skewness	Kurtosis
CCSE T1	60.02±8.62	60.00	38.75	82.81	0.06	–0.01
CCSE T2	68.98±8.34	68.91	43.75	90.00	–0.18	0.44
CCSE T3	77.62±7.72	78.59	50.00	93.75	–0.65	0.56
CPX T1	65.83±5.88	66.16	46.39	79.61	–0.82	1.59
CPX T2	78.44±4.85	79.23	65.52	87.69	–0.47	–0.45
PT T1	41.39±13.89	40.00	8.00	71.33	0.11	–0.61
PT T2	57.82±10.96	58.00	25.33	81.33	–0.17	–0.31
PT T3	70.18±9.00	71.33	38.67	93.33	–0.38	0.80

All examination scores are presented on a converted 100-point scale. SD, standard deviation; CCSE, comprehensive clinical science examination; CPX, clinical performance examination; PT, progress test.

**Table 3.** Correlations between study variables at T1, T2, and T3

Variable	1	2	3	4	5	6	7	8
1. CCSE T1	1							
2. CCSE T2	0.666^a)^	1						
3. CCSE T3	0.596^a)^	0.780^a)^	1					
4. CPX T1	0.505^a)^	0.440^a)^	0.483^a)^	1				
5. CPX T2	0.349^a)^	0.254^b)^	0.393^a)^	0.489^a)^	1			
6. PT T1	0.623^a)^	0.380^a)^	0.408^a)^	0.349^a)^	0.402^a)^	1		
7. PT T2	0.774^a)^	0.680^a)^	0.655^a)^	0.422^a)^	0.297^b)^	0.609^a)^	1	
8. PT T3	0.607^a)^	0.665^a)^	0.707^a)^	0.410^a)^	0.338^a)^	0.534^a)^	0.699^a)^	1

CCSE, comprehensive clinical science examination; CPX, clinical performance examination; PT, progress test.

^a)^Correlation is significant at P<0.001. ^b)^Correlation is significant at P<0.01.

**Table 4.** Model fit indices for the 8 autoregressive cross-lagged models

Model	χ^2^	df	CFI	TLI	SRMR	Δχ^2^	Δdf	ΔCFI
Model 1	6.604	6	0.999	0.995	0.016	-	-	-
Model 2	6.827	7	1.000	1.001	0.017	0.170	1	0.001
Model 3	10.925	8	0.994	0.981	0.025	3.901^b)^	1	–0.006
Model 4	12.388	9	0.994	0.980	0.029	1.255	1	0.000
Model 5	16.766	10	0.987	0.964	0.043	6.015^b)^	1	–0.007
Model 6	23.307	11	0.977	0.941	0.045	7.388^a)^	1	–0.010
Model 7	25.262	12	0.975	0.942	0.060	2.101	1	–0.002
Model 8	27.647	13	0.972	0.941	0.058	2.925	1	–0.003

df, degrees of freedom; CFI, comparative fit index; TLI, Tucker-Lewis index; SRMR, standardized root-mean square residual.

^a)^Correlation is significant at P<0.01. ^b)^Correlation is significant at P<0.05.

## Footnotes

**Data availability**
Data files are available from https://doi.org/10.7910/DVN/F8SI8E Dataset 1. Raw observational data.

## Supplementary materials

Supplement 1. R code for the analyses.

Supplement 2. Audio recording of the abstract.

## References

[1] Lockyer J, Carraccio C, Chan MK, Hart D, Smee S, Touchie C, Holmboe ES, Frank JR; ICBME Collaborators (2017). Core principles of assessment in competency-based medical education. Med Teach.

[2] Neeley SM, Ulman CA, Sydelko BS, Borges NJ (2016). The value of progress testing in undergraduate medical education: a systematic review of the literature. Med Sci Educ.

[3] Fitz M, Adams W, Heincelman M, Haist S, Whelan K, Cox L, Cao UT, Hingle S, Raff A, Houghton B, Fitzpatrick J, Nall R, Foster J, Appelbaum J, Grum C, Donovan A, Kiken S, Abraham R, Hlafka M, Miller C, Bansal S, Paauw D, Lai CJ, Pincavage A, Agarwal G, Burns C, Holzer H, Lappe K, John V, Barker B, Mingioni N, Rao D, Zakowski L, Chakraborti C, Williams W, Kelly W (2022). The impact of internal medicine clerkship characteristics and NBME subject exams on USMLE Step 2 clinical knowledge exam performance. J Gen Intern Med.

[4] Wearn A, Bindra V, Patten B, Loveday BP (2023). Relationship between medical programme progress test performance and surgical clinical attachment timing and performance. Med Teach.

[5] Brallier I, Mahmood S, Grotkowski K, Taylor J, Zdon M (2021). Does surgical observed structured clinical exam (OSCE) predict clerkship grade, shelf exam scores, and preceptor clinical evaluation?. Am J Surg.

[6] Selig JP, Little TD, Laursen B, Little TD, Card NA (2012). Handbook of developmental research methods.

[7] Rosseel Y (2012). lavaan: an R package for structural equation modeling. J Stat Softw.

[8] Kaufman DM, Swanwick T, Forrest K, O’Brien BC (2018). Understanding medical education: evidence, theory, and practice.

[9] Cecilio-Fernandes D, Bicudo AM, Hamamoto Filho PT (2021). Progress testing as a pattern of excellence for the assessment of medical students’ knowledge: concepts, history, and perspective. Medicina (Ribeirão Preto).

[10] Griffin B, Bayl-Smith P, Hu W (2018). Predicting patterns of change and stability in student performance across a medical degree. Med Educ.

[11] Gorlich D, Friederichs H (2021). Using longitudinal progress test data to determine the effect size of learning in undergraduate medical education: a retrospective, single-center, mixed model analysis of progress testing results. Med Educ Online.

[12] van Wijk EV, van Blankenstein FM, Janse RJ, Dubois EA, Langers AM (2024). Understanding students' feedback use in medical progress testing: a qualitative interview study. Med Educ.

[13] Henrique-Sanches BC, Cecilio-Fernandes D, Costa RR, Almeida RG, Etchegoyen FF, Mazzo A (2024). Implications of clinical simulation in motivation for learning: scoping review. Einstein (Sao Paulo).

[14] Dion V, St-Onge C, Bartman I, Touchie C, Pugh D (2022). Written-based progress testing: a scoping review. Acad Med.

[15] Hassan EM, Kannan LS, Al Suliman A, Lewis S, Al Majed H, Al Abdullatif S, Daniel S, Al Matouq JA (2024). Enhancing academic monitoring: examining the correlation between the results of progress test and the cumulative grade point averages of nursing students. Educ Adm Theory Pract.

